# Circulating microRNA signature of genotype‐by‐age interactions in the long‐lived Ames dwarf mouse

**DOI:** 10.1111/acel.12373

**Published:** 2015-07-14

**Authors:** Berta Victoria, Joseph M. Dhahbi, Yury O. Nunez Lopez, Lina Spinel, Hani Atamna, Stephen R. Spindler, Michal M. Masternak

**Affiliations:** ^1^ Burnett School of Biomedical SciencesCollege of MedicineUniversity of Central Florida6900 Lake Nona Blvd.OrlandoFL32827USA; ^2^ Department of BiochemistryUniversity of California at RiversideRiversideCA92521USA; ^3^ Center for GeneticsChildrens Hospital Oakland Research InstituteOaklandCA94609USA; ^4^ Translational Research Institute for Metabolism and DiabetesFlorida Hospital301 E. Princeton StreetOrlandoFL2804USA; ^5^ Department of Medical EducationCalifornia Northstate UniversityElk GroveCAUSA; ^6^ Department of Head and Neck SurgeryThe Greater Poland Cancer Centre15 Garbary St.61‐866PoznanPoland

**Keywords:** aging, circulating miRNAs, dwarf mouse, sequencing, sncRNAs, tRNA halves

## Abstract

Recent evidence demonstrates that serum levels of specific miRNAs significantly change with age. The ability of circulating sncRNAs to act as signaling molecules and regulate a broad spectrum of cellular functions implicates them as key players in the aging process. To discover circulating sncRNAs that impact aging in the long‐lived Ames dwarf mice, we conducted deep sequencing of small RNAs extracted from serum of young and old mice. Our analysis showed genotype‐specific changes in the circulating levels of 21 miRNAs during aging [genotype‐by‐age interaction (GbA)]. Genotype‐by‐age miRNAs showed four distinct expression patterns and significant overtargeting of transcripts involved in age‐related processes. Functional enrichment analysis of putative and validated miRNA targets highlighted cellular processes such as tumor suppression, anti‐inflammatory response, and modulation of Wnt, insulin, mTOR, and MAPK signaling pathways, among others. The comparative analysis of circulating GbA miRNAs in Ames mice with circulating miRNAs modulated by calorie restriction (CR) in another long‐lived mouse suggests CR‐like and CR‐independent mechanisms contributing to longevity in the Ames mouse. In conclusion, we showed for the first time a signature of circulating miRNAs modulated by age in the long‐lived Ames mouse.

## Introduction

MicroRNAs (miRNAs) are small noncoding RNAs (sncRNAs) known to mediate different cellular functions through post‐transcriptional regulation (Djuranovic *et al*., 2011). Mammalian protein‐coding genes represent conserved targets of miRNAs, while each miRNA can target multiple mRNAs (Friedman *et al*., 2009). MicroRNAs may play important role in aging processes by post‐transcriptional regulation of gene expression and modulation of physiological changes. Beside cellular miRNAs, it is well known that miRNAs can be released into the bloodstream and circulate within extracellular spaces [reviewed in Dhahbi (2014a)]. Some circulatory miRNAs may be packaged in lipid vesicles or complexed with high density lipoproteins particles or RNA‐binding proteins (Vickers *et al*., 2011).

More recently, deep sequencing technologies allowed the identification of new types of small RNAs derived from the processing of already known sncRNAs including tRNA (Rutjes *et al*., 1999; Rother & Meister, 2011; Sobala & Hutvagner, 2011). There is evidence implicating these derivatives of sncRNAs in cell‐to‐cell communication both in normal biology and in disease states (Cortez *et al*., 2011; Hoy & Buck, 2012; Shah & Calin, 2012; Turchinovich *et al*., 2012; Kosaka *et al*., 2013). Our deep sequencing studies of serum/plasma have consistently detected tRNA‐derived RNAs of size 30–33 nt (Dhahbi *et al*., 2013a,b). Intracellular tRNA‐derived small RNAs are classified into two types based on their size (Sobala & Hutvagner, 2011; Martens‐Uzunova *et al*., 2013): tRNA halves with size of 30–40 nt produced by cleavage of mature tRNAs, and shorter tRNA‐derived fragments (tRFs) of size 18–22 nt produced from both mature and pre‐tRNAs by Dicer or RNase Z (Thompson *et al*., 2008; Cole *et al*., 2009; Fu *et al*., 2009; Lee *et al*., 2009; Thompson & Parker, 2009a; Pederson, 2010; Sobala & Hutvagner, 2011). The tRNA halves class includes 5′‐ and 3′‐tRNA halves that were first observed in stressed cultured cells where they are produced by cleavage of tRNAs near or at the anticodon loop with the ribonuclease Rny1 in *Saccharomyces cerevisiae* (Thompson & Parker, 2009b) and Angiogenin in higher eukaryotes (Fu *et al*., 2009; Yamasaki *et al*., 2009). They were later observed in unstressed human cells (Kawaji *et al*., 2008; Fu *et al*., 2009). However, their levels in resting cells are very low and often increase significantly only during stress conditions (Saikia *et al*., 2012). Our group and others detected tRNA‐derived small RNAs circulating in mouse and human bloodstream (Meiri *et al*., 2010; Dhahbi *et al*., 2013a,b, 2014; Dhahbi, 2014a). They were later found in rat and monkey serum at levels higher than miRNAs (Zhang *et al*., 2014), and also in another biological fluid, human semen (Vojtech *et al*., 2014). The tRNA‐derived small RNAs found in serum/plasma originate mostly from the 5′ end of distinct subsets of tRNAs (*5′ tRNA halves*) and are as abundant as miRNAs (Dhahbi *et al*., 2013a,b; Dhahbi, 2014b). The preponderance of 5′‐ over 3′‐end fragments may reflect functional and/or stability differences. The shorter tRFs were not detected at the sequencing depths we used in our studies of circulating sncRNAs.

Changes in gene expression are strongly associated with regulation of aging and longevity (Dhahbi *et al*., 2004, 2007). One of the most powerful interventions that can extend mammalian longevity is calorie restriction (CR) and at the same time CR alters the expression pattern of genes that are affected by aging (Masternak *et al*., 2004, 2005). Beside its known alterations of gene expression, CR can also modulate the pattern of circulating miRNAs in mice (Dhahbi *et al*., 2013d). However, there are known genetic interventions that also alter lifespan of mice. Suppression of growth hormone (GH) and insulin like growth factor 1 (IGF‐1) signaling pathway provides the most significant lifespan extension on animal models (Bartke *et al*., 2001; Bartke & Brown‐Borg, 2004). One well‐established model for aging and longevity research is the Ames dwarf mouse (df/df; Bartke *et al*., 2001; Bartke & Brown‐Borg, 2004; Masternak *et al*., 2004; Dhahbi *et al*., 2007; Bates *et al*., 2010; Menon *et al*., 2014). This mutant mouse is characterized by the deficiency in three pituitary hormones including GH, prolactin, and thyrotropin due to homozygous, spontaneous mutation in the prophet of pituitary factor 1 (Prop1), a transcription factor responsible for pituitary development. Due to GH deficiency, dwarf mice have severely suppressed IGF‐1 levels in circulation (Masternak *et al*., 2004; Menon *et al*., 2014). Importantly, df/df mice live between 40% and 60% longer than their normal littermates (Bartke *et al*., 2001; Bartke & Brown‐Borg, 2004). Beside extended lifespan, these animals are also characterized by extended health span. Ames dwarf mice are also highly insulin sensitive and have improved glucose tolerance, enhanced memory and learning skills as they age and are protected from cancer (Kinney *et al*., 2001; Ikeno *et al*., 2003; Menon *et al*., 2014). A recent study with short‐term, early‐life GH replacement therapy demonstrated that supplementing GH to df/df mice shortens their lifespan to the equivalent range of normal littermates (Panici *et al*., 2010). Regardless of the deficiency in three different hormones in df/df mice, GH seems to be a main regulator of lifespan in healthy animals. These hormonal alterations may play important role in the patterns of circulating miRNAs reported in the present study, as it has been previously shown that groups of miRNA may regulate or are regulated by endocrine signals (Poy *et al*., 2004). We previously showed altered patterns and regulations of liver miRNA in Ames dwarf mice (Bates *et al*., 2010); however, in the present study we examined circulating miRNA in young and old df/df and normal mice. 

## Results

### Analysis of circulating small RNA sequencing reads

**Figure 1:**
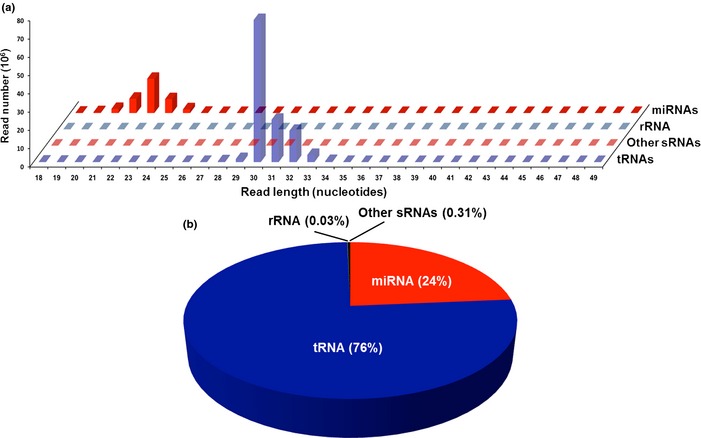
Length distribution and annotation of small RNAs circulating in mice serum. Two major small RNA peaks were detected in the serum from the studied mice: at 20–24 nt, consistent with the size of miRNAs, and at 30–33 nt consisted of reads mapping to tRNA genes (a). A total of 76% and 24% of the total reads mapped to the mouse small noncoding RNAs were derived from tRNAs and miRNAs, respectively (b).

To investigate potential relationships among circulating small RNAs and aging‐related processes modulated in the long‐lived Ames dwarf (df/df) mice, we conducted deep sequencing of small RNAs extracted from serum of young and old mice. We detected two major small RNA peaks. The size distribution of the mapped reads revealed an expected peak at 20–24 nt, consistent with the size of miRNAs. The second peak occurred at 30–33 nt and consisted of reads mapping to tRNA genes. This peak represents a class of tRNA‐derived fragments (tRNA halves) previously described (Dhahbi *et al*., 2013b; Fig. 1a). Further analysis showed that 76% and 24% of the total reads that mapped to the mouse genome were derived from tRNAs and miRNAs, respectively (Fig. 1b).

### The abundance of circulating miRNAs is differentially modulated by age in N and df/df mice

In an effort to identify longevity‐associated miRNAs, we assessed differential expression between df/df mice and aged‐matched N controls at two different ages. This approach allows the identification of miRNAs that exhibit significant genotype‐by‐age (GbA) interaction. Our analysis detected 21 circulating miRNAs exhibiting significant GbA interaction with *P* < 0.05 and false discovery rate (FDR) < 0.10 (Table 1). Our analysis also indicated additional 21 circulating miRNAs exhibited significant GbA with *P* < 0.05; however, they were not included in our discussion because of FDR > 0.10 (0.10 < FDR < 0.38; Table S1, Supporting information). A group of 17 miRNAs remained unchanged during aging in the df/df mice but changed expression in the N mice: three of them (Table 1, pattern A) increased and 14 (Table 1, pattern B) decreased their respective circulating levels. A second group of four miRNAs increased expression with age in the df/df mice but not in the N mice, where three of them (Table 1, pattern C) showed significantly reduced levels in the old N animals and one (Table 1, pattern D) did not change with age. We did not find miRNAs downregulated by age in the df/df mice at the chosen level of statistical significance (Table 1).

**Table 1:** Circulating miRNAs exhibiting significant genotype‐by‐age (GbA) in df/df and N mice, with four distinct expression patterns

Mus musculus miRNA^a^	Family	CPM^b^	Age effect in Normal^c^			Age effect in df/df^c^			GbA interaction^d^			Pattern
			FC	*P*‐value	FDR	FC	*P*‐value	FDR	FC	*P*‐value	FDR	
miRNAs whose serum abundance increases with age in normal mice but remains unchanged in df/df mice												
mmu‐miR‐5107‐5p	mir‐5107	8	9.9	0.000	0.000	1.3	0.916	1.000	−7.8	0.002	0.079	A
mmu‐miR‐146a‐5p	mir‐146	165	5.4	0.000	0.000	1.2	0.812	1.000	−4.4	0.003	0.096	
mmu‐miR‐342‐5p	mir‐342	60	3.0	0.000	0.001	1.1	0.920	1.000	−2.8	0.002	0.084	
miRNAs whose serum abundance decreases with age in normal mice but remains unchanged in df/df mice												
mmu‐miR‐344d‐3‐5p	mir‐344	3	−7.1	0.000	0.004	2.3	0.329	0.788	16.5	0.001	0.058	B
mmu‐miR‐376c‐5p	mir‐368	5	−30.0	0.000	0.000	−1.7	0.248	0.753	17.4	0.002	0.074	
mmu‐miR‐136‐5p	mir‐136	156	−15.4	0.000	0.000	−1.6	0.205	0.731	9.7	0.002	0.079	
mmu‐miR‐411‐5p	mir‐379	33	−19.2	0.000	0.000	−1.6	0.249	0.753	12.1	0.002	0.074	
mmu‐miR‐344d‐1‐5p	mir‐344	3	−8.4	0.000	0.004	1.5	0.330	0.788	12.3	0.001	0.058	
mmu‐miR‐410‐5p	mir‐154	58	−8.3	0.000	0.000	−1.3	0.434	0.875	6.3	0.002	0.076	
mmu‐miR‐369‐5p	mir‐154	5	−31.8	0.000	0.000	−1.5	0.436	0.876	21.4	0.000	0.058	
mmu‐miR‐154‐5p	mir‐154	7	−63.9	0.000	0.000	−1.5	0.435	0.876	41.6	0.001	0.059	
mmu‐miR‐540‐5p	mir‐540	6	−23.8	0.000	0.000	−1.3	0.526	0.958	17.7	0.001	0.058	
mmu‐miR‐127‐5p	mir‐127	498	−19.4	0.000	0.000	−1.2	0.496	0.925	16.0	0.000	0.058	
mmu‐miR‐449a‐5p	mir‐449	3	−4.0	0.000	0.000	1.1	0.935	1.000	4.3	0.001	0.059	
mmu‐miR‐381‐5p	mir‐154	77	−15.2	0.000	0.000	−1.1	0.790	1.000	13.7	0.001	0.058	
mmu‐miR‐344d‐3p	mir‐344	3	−4.7	0.000	0.004	1.7	0.330	0.788	8.1	0.001	0.058	
mmu‐miR‐195a‐5p	mir‐15	56	−3.2	0.000	0.000	−1.0	0.972	1.000	3.2	0.001	0.058	
miRNAs whose serum abundance decreases with age in normal mice but increases in df/df mice												
mmu‐miR‐34c‐5p	mir‐34	28	−5.1	0.000	0.003	4.1	0.005	0.118	20.9	0.000	0.004	C
mmu‐miR‐34b‐5p	mir‐34	7	−2.7	0.018	0.087	3.3	0.008	0.145	9.0	0.000	0.058	
mmu‐miR‐344d‐2‐5p	mir‐344	3	−4.8	0.000	0.004	2.4	0.329	0.788	11.3	0.001	0.058	
miRNAs whose serum abundance does not change with age in normal mice but increases in df/df mice												
mmu‐miR‐592‐5p	mir‐592	10	1.2	0.906	1.000	20.8	0.000	0.000	17.3	0.000	0.058	D

a Names of mature miRNAs of Mus musculus in miRbase v.21 (GRCm38) that match precursor sequences predicted by miRDeep2, and have a randfold *P*‐value < 0.05.

b Average known miRNA read counts‐per‐million computed over all libraries and taking into account the estimated dispersions and the library sizes.

c Fold change, *P*‐value and false discovery rate (FDR) for differential abundance were computed by edgeR from pairwise comparisons for each miRNA between the young and old groups within each of the indicated genotypes. Age effect is considered significant if ¦FC¦ ≥ 1.5 and *P*‐value < 0.05.

d Genotype‐by‐age interaction. Fold change of the interaction represents the difference of the fold changes between df/df and N with respect to age. Only miRNAs with an interaction ¦FC¦ > 1.5 and FDR < 0.1 are reported.

### Circulating GbA miRNAs are not enriched with tissue‐specific miRNAs

The origin of cell‐free circulating miRNAs is unclear, but they must be released into the animal circulation by specific cells/tissues either due to active mechanisms of miRNA secretion (e.g., release of miRNA‐containing exosomes; Weilner *et al*., 2013) or spillover of cytoplasmic contents (e.g., due to cell demise; Farr *et al*., 2013). Using mouse tissue‐specific miRNA signatures recently described by Guo *et al*. (2014), we assessed whether our GbA miRNA signature was significantly enriched in tissue‐specific miRNAs. No significant enrichment for kidney‐, heart‐, or brain‐specific miRNAs was detected; therefore, we rule out the spillover of cytoplasmic contents from these tissues. Rather, these results suggest the release of miRNAs into the circulation possibly through an active secretion mechanism. This also rules out the possibility of contamination of the circulating GbA miRNA signature with miRNAs from heart tissue damaged during the cardiac puncture.

### Pathways relevant to aging are associated with circulating miRNAs that show significant GbA interaction between N and df/df mice

To functionally characterize the distinct groups of miRNAs exhibiting significant GbA interaction, we identified gene ontology (GO) categories and cellular pathways significantly enriched in our lists of predicted target genes (Tables S2–S5, Figs S2–S5, Supporting information). We found that the targets of GbA miRNAs from each pattern are enriched for common sets of biological processes including cell–cell signaling, neuronal transmission, and apoptosis among others. They were also enriched in multiple cancer pathways and signaling driven most commonly by MAPK and Wnt, as well as Erb, insulin, notch, hedgehog, and mTOR among others.

### Analysis of GbA miRNA–mRNA interaction network identifies subsets of overtargeted genes

**Figure 2:**
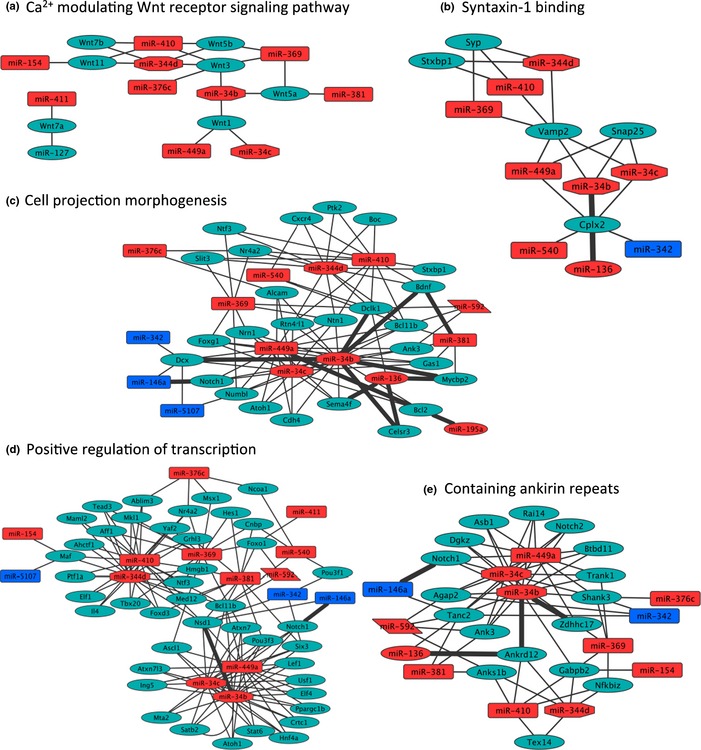
Gene‐by‐age interaction miRNA–mRNA network analysis. Five subnetworks of miRNA‐overtargeted transcripts enriched for biological processes and molecular functions relevant to aging are presented. (a) Calcium modulating Wnt receptor signaling pathway; (b) SNARE/syntaxin binding; (c) cell projection morphogenesis; (d) positive regulation of transcription; (e) genes containing ankyrin repeats. Noticeable is a complex pattern of hubs and interhub interactions that underscore a relevant cross talk among pathways. The genotype‐by‐age (GbA) miRNA–mRNA interaction network was constructed in Cytoscape 3.0.2 software as described in Materials and methods. Red/blue boxes: positive/negative GbA miRNAs. Cyan ovals: mRNAs (predicted to be downregulated).

The analysis of all predicted targets has the inconvenience of considering a highly dimensional set of interactions due to the large number of software‐predicted miRNA targets, which are not all biologically relevant. To identify a more relevant subset of predicted targets, we conducted overrepresentation analysis of all GbA miRNA‐targeting events on each predicted target. We identified 729 genes significantly overtargeted by GbA miRNAs (Table S6, *P* < 0.05 and FDR < 0.10). Functional annotation clustering performed on this gene set, using the highest stringency settings on DAVID Bioinformatic Database, identified multiple enriched clusters of biological processes and protein domains that characterize the overtargeted gene set (Table S7). These clusters highlighted overtargeted genes involved in Wnt receptor signaling, cell projection morphogenesis/axonogenesis, positive regulation of transcription, positive regulation of biosynthetic processes, syntaxin/SNARE binding, and genes containing ankyrin repeats. Figure 2 shows multiple miRNA–mRNA subnetworks of relevant regulatory relationships among GbA miRNAs and the functionally enriched overtargeted genes. Two main interaction hubs are highlighted by the network approach: one centered at miR‐34b/miR‐34c/miR‐449a and another at miR‐344d/miR‐410/miR‐369. These miRNA hubs underscore the major roles played by pattern B and pattern C miRNAs during aging in df/df mice.

### Common and specific mechanisms may drive age‐associated changes in circulating miRNAs in both long‐lived df/df mice and in B6C3F1 mice under caloric restriction

**Figure 3:**
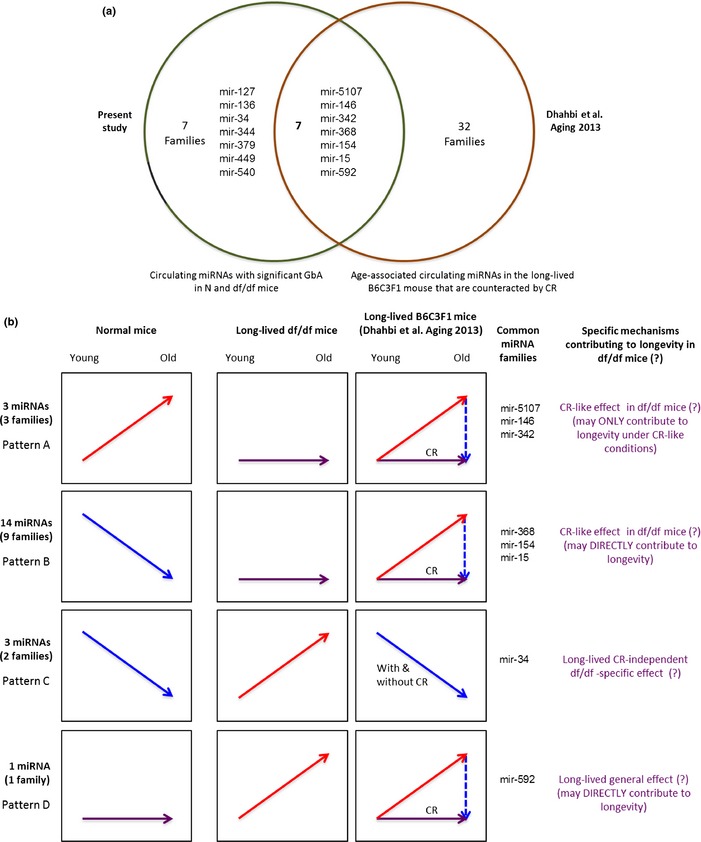
Comparative analysis of circulating genotype‐by‐age (GbA) miRNAs in mice. (a) Venn diagram of common miRNA families between this work and the study of Dhahbi *et al*. (2013c). (b) GbA miRNAs in N and df/df mice exhibited four different types of expression patterns (left and middle panel). Many miRNAs circulating in the long‐lived B6C3F1 mouse (within common GbA miRNA families) are increased with age, and this effect can be antagonized by calorie restriction (CR; right panel).

To gain insights on the effect of aging on circulating miRNAs, we compared the circulating miRNAs exhibiting significant GbA in N and df/df mice (data from the present study) with changes in circulating miRNAs reported for the hybrid long‐lived B6C3F1 mouse (Dhahbi *et al*., 2013d). The comparison showed that 50% (7/14) of circulating miRNA families that show a GbA phenotype in our study are also modulated by age and CR in the B6C3F1 mice (Venn diagram shown in Fig. 3a). Although mice of distinct gender were used in these studies (df/df females vs. calorie‐restricted B6C3F1 males), the impact on our comparison should be limited because we focused on the common miRNA families detected by the two studies. In addition, differences in miRNA expression between mice of distinct gender appear to occur in young mice. In this regard, Kwekel *et al*. (2010) reported that in the rat liver, sex differences in gene expression extended until middle age and then expression profile of male dramatically converged to that of female during later aging. Because Dhahbi and collaborators used only old CR male mice to compare to old and young normal controls, the expression profile differences reported by these authors are expected to be equivalent to those from old CR females.

All three miRNA families within the pattern A (i.e., miR‐5107, miR‐146, miR‐342), which depict a negative GbA in df/df mice, were common between df/df and CR B6C3F1 mice. Their circulating levels increased with age in both the N and the hybrid B6C3F1 mice. However, these miRNA levels did not change with age in the hybrid mice under CR, similar to our df/df mice (Fig. 3b, upper panel). This suggests that GbA pattern A miRNAs may not be directly contributing to long life under normal conditions, but could be improving aging under CR (experimentally induced in the hybrid mouse study) or CR‐like (naturally and partially activated in the df/df mouse) conditions. In addition, these mice shared three other miRNA families within the GbA pattern B. This group displays positive GbA, given by circulating levels that decrease during aging in N mice but increase in B6C3F1 mice and were not affected by age in either df/df mice or B6C3F1 mice under CR (Fig. 3b, second panel). This suggests that pattern B miRNAs are contributing to df/df longevity through CR‐like mechanisms.

Members of pattern C miRNA family mir‐34 were increased in old df/df mice, while they were decreased in both N and B6C3F1 old mice. The positive‐GbA interaction displayed by the mir‐34 family in df/df mice contrasts with its behavior in the B6C3F1 long‐living hybrid mice. Similar to our normal aging mice, circulating miR‐34 levels decreased in the old hybrid mice and this expression pattern was not modified by CR (Fig. 3b, pattern C). This pattern suggests a longevity‐related mechanism that could be independent from CR conditions and specific for df/df mice. Pattern D circulating miR‐592‐5p (family mir‐592) was commonly upregulated in old B6C3F1 and in df/df mice, but did not change in B6C3F1 mice under CR as in N mice (Fig. 3b, bottom panel). This pattern suggests a possible common mechanism directly contributing to the long‐lived phenotype in df/df and B6C3F1 mice.

### The abundance of circulating 5′ tRNA halves is differentially modulated by age in N and df/df mice

We have previously shown that CR mitigated age‐related changes in circulating levels of 5′ tRNA halves in mice (Dhahbi *et al*., 2013b). Dwarfism, similar to CR, can delay, prevent, or reverse many age‐associated changes in physiological parameters (Tsuchiya *et al*., 2004; Ikeno *et al*., 2013). Thus, we assessed differential abundance of circulating 5′ tRNAs halves between df/df mice and aged‐matched N controls at two different ages. The analysis suggest that both aging and dwarfism are associated with alterations in the circulating levels of 5′ tRNA halves derived from specific tRNA isoacceptors (Table S8, *P* < 0.05 and FDR < 0.28). In normal mice, aging is associated with a decrease in the circulating levels of 5′ tRNA halves derived from tRNA‐Cys (GCA) and tRNA‐Lys (CTT), and an increase in the circulating levels of 5′ tRNA halves derived from tRNA‐His(GTG) and tRNA‐Asp(GTC); however, in df/df mice the circulating levels of these 5′ tRNA halves remained unchanged with age. Dwarfism is associated with a decrease in the circulating levels of 5′ tRNA halves derived from several tRNA‐Pro isoacceptors while aging had no effect. However, because of the FDR > 0.10 these data need more intense studies and additional future evaluations to determine potential important biological functions.

## Discussion

Our study found that a majority of circulating GbA miRNAs shows a positive‐GbA interaction in df/df mice as compared to normal controls (Table 1). Recently, Dhahbi *et al*. (2013d) reported a similar behavior in another mouse model of extended lifespan. These authors found only three downregulated miRNAs in a total of 48 differentially expressed circulating miRNAs in old long‐lived B6C3F1 hybrid mice (as compared to young counterparts). This suggests a general trend for higher circulating miRNA levels in long‐lived mice. In contrast, a general trend for reduction in miRNA levels is observable during aging in normal mouse. Based on these observations, we could speculate that GbA miRNAs play an active role in the downregulation of transcripts that would otherwise wreak havoc cellular homeostasis as the organism age. We found that the extended lifespan in the df/df mice appear regulated by miRNAs that act through CR‐like and/or CR‐independent mechanisms. Relevant to these findings are the observations by Ikeno *et al*. (2013) that df/df and CR mice exhibit anti‐aging effects through both independent and common mechanisms. The authors suggested that common mechanisms should be driven by changes in the endocrine system, but were unable to identify clues for the CR‐independent mechanisms. Our study suggests that such CR‐independent mechanisms involve the action of miRNAs from patterns C and D (Fig. 3b, bottom panels).

The collection of 21 GbA miRNAs found in our study comprises 14 miRNA families. Three of these families are represented by more than one miRNA (Table 1). Notably, family miR‐154 is represented by four members (in pattern B, positive GbA). Previous studies associated members of this family with cell signaling and tumor suppression functions in both mice and humans (Zheng *et al*., 2012; Formosa *et al*., 2013). Levels of these tumor suppressor miRNAs diminished in the old N mice but remained unchanged in df/df mice. The relative higher levels of these tumor‐suppressing miRNAs in old df/df mice (as compared to old N mice) suggest a reduced risk of or delayed progression through cancer. Notably, Ikeno *et al*. (2003) reported that fatal neoplastic disease occurred later in life in df/df mice, and the severity of lung adenocarcinoma was lower compared with wild‐type littermates. Three members of the mir‐344 family were distributed in two distinct positive‐GbA patterns (i.e., B and C). These miRNAs have been associated with regulation of adipogenesis through the Wnt signaling pathway (Chen *et al*., 2014). Importantly, we found that miR‐344 is a hub miRNA that seems to play a key role in the regulation of GbA miRNA–mRNA networks. Our analysis revealed that overtargeted genes are indeed significantly enriched for Wnt signaling molecules (Fig. 2a, Tables S6 and S7). We reason that circulating miRNAs may be downregulating Wnt signaling in target tissues prone to uncontrolled cell proliferation and development of cancer. Indeed, several genes in the Wnt/β‐catenin signaling network have been found negatively correlated with age and tightly regulated by miRNAs (e.g., miR‐34 family; Kim *et al*., 2011).

Notably, we demonstrated in this study that two members of the mir‐34 family (miR‐34b and miR‐34c) are important hub miRNAs and central regulators of functionally enriched subnetworks (Fig. 2). These miRNAs are downregulated with age in N mice, while increased in df/df mice (pattern C, positive GbA). Pattern C miRNAs seem to contribute to the df/df mouse longevity by a CR‐independent mechanism. Recent reports demonstrate tumor suppressor (Wu *et al*., 2013) and neuroprotective (Minones‐Moyano *et al*., 2011) functions for these miRNAs. Wu and colleagues found downregulation of miR‐34c‐3p/5p in glioma tissue. Overexpression of these two miRNAs in glioma cell lines inhibited the cell proliferation, cell cycle changes, apoptosis, and cell invasion (Wu *et al*., 2013). We found that predicted targets of the pattern C GbA miRNAs are significantly enriched in the MAPK signaling pathway (Table S4). MAPK signaling pathways are well known to play important roles in cancer and sensing/transducing stress signals. A neuroprotective role was also suggested for miR‐34b/c because they were broadly downregulated in Parkinson's disease brains, occurring early in the course of the disease and underlying mitochondrial dysfunction and oxidative stress (Minones‐Moyano *et al*., 2011). Our functional analysis of pattern C miRNA‐overtargeted genes uncovered enrichment for several neurodevelopmental and synaptic signaling‐related biological processes (Table S4). Similarly, we found that predicted targets of the pattern D GbA miRNA are significantly enriched for Wnt signaling and neurodevelopmental biological processes (Table S5).

In contrast with pattern B, C, and D, the pattern A miRNAs show a distinctive negative GbA interaction. These miRNAs do not change expression during aging in df/df mice, but increase in N mice (Table 1). This group seems to contribute to inflammation during normal aging. A member of this group, miR‐342‐5p, was previously found upregulated in early atherosclerotic lesions in Apoe(−/−) mice (Wei *et al*., 2013). That study showed that miR‐342‐5p promotes inflammatory macrophage activation through an Akt1 and miR‐155‐dependent pathway during atherosclerosis (Wei *et al*., 2013). In addition, two of the pattern A miRNAs (i.e., miR‐342 and miR‐146) have been associated with inflammation, proliferation, and cancer (Fayyad‐Kazan *et al*., 2013).

Overtargeting of Slc2a1 (Glut1) by miR‐410 and miR‐344d (pattern B and C) may also contribute to reduce inflammation in old df/df mice by maintaining this glucose transporter downregulated. Recent reports indicate that glucose is the primary fuel metabolized in proinflammatory macrophages (Freemerman *et al*., 2014). These authors found that adipose tissue macrophages expressing high levels of surface Slc2a1 contained higher levels of cytokines, suggesting that Slc2A1 correlates with *in vivo* inflammation. Importantly, there is strong association of aging with chronic low‐grade inflammatory activity, which may progress to chronic systemic inflammation and cause organ‐specific illness with increased risk of mortality (Masternak & Bartke, 2012). Another gene overtargeted by hub GbA miRNAs miR‐410 and miR‐344d is Hdac1 (Table S6). It is reported that Cugbp1‐mediated translational elevation of Hdac1 is one of the key events conducing to epigenetic changes in the liver of old mice, which lead to the development of age‐associated dysfunction of the liver [reviewed in Jones *et al*. (2012)]. Elevated activity of these miRNAs in df/df mice may therefore contribute to reduce systemic inflammation and improve health span.

Interestingly, two of the GbA miRNA families (miR‐146 and miR‐92) were found represented in inflammatory microvesicles associated with metabolic and cardiovascular disease (Hulsmans & Holvoet, 2013). This finding supports a role for these miRNAs in nonautonomous expression regulation by acting at distant locations (e.g., peripheral tissues reached through the circulation).

A counterintuitive but important finding from our analysis is the overtargeting of FoxO1 by several circulating positive‐GbA miRNAs, which should reduce Foxo1 levels in particular tissues. In general, activation of Foxo1 has been associated with lifespan extension in animal models by facilitating defense against oxidative and other cellular stressors (Murakami, 2006). This effect appears mediated by the interaction of FoxO proteins with β‐catenin, with the latter being diverted from TCF and therefore attenuating the canonical Wnt pathway (MacDonald *et al*., 2009). On the other hand, this FoxO‐driven molecular switch has also been found to mediate a pathogenic mechanism for osteoporosis by decreasing the number of matrix‐synthesizing osteoblasts and the amount of bone mass. Notably, mice lacking all three redundant FoxO genes in bipotential progenitors of osteoblast and adipocytes exhibited significant bone gain that remained in the old mice, and decreased number of adipocytes in the aged bone marrow (Iyer *et al*., 2013). Moreover, overexpression of FoxO1 in skeletal muscle reduced muscle mass and impaired glycemic control (Kamei *et al*., 2004). Furthermore, FoxOs appear to contribute to impaired β‐cell compensation of insulin resistance, increased hepatic glucose production, and hyperlipidemia, which are critical pathogenic processes conducing to type 2 diabetes and metabolic syndrome (Manolagas & Almeida, 2007). Mounting evidence including the rescue of insulin sensitivity on FoxO1 haploinsufficient mice and the induction of diabetes in FoxO1 gain‐of‐function mutant mouse shed light on these pathogenetic roles of FoxO proteins (Nakae *et al*., 2002). We hypothesize that circulating GbA miRNAs that target FoxO1, will hone in the bone, skeletal muscle, and pancreas.

Another interesting result from the functional annotation analysis of GbA miRNA‐overtargeted genes was the enrichment for genes containing ankyrin repeats (Table S7). Notably, Ank3 (ankyrin G), a gene overtargeted by GbA miRNAs in df/df mice, was found overexpressed in Hutchinson‐Gilford progeria syndrome (HGPS; MIM 176670), a rare disease characterized by accelerated aging (Wang *et al*., 2006). Overexpression of ankyrin G was secondary to the LMNA mutation that is the primary insult in HGPS. Our result suggests that increased levels of ankyrin G might contribute to the progression to early senescence and that miRNAs can regulate this process to beneficially impact aging. Another set of proteins (band 3 proteins), which perform ankyrin binding functions, play a key role in the generation of senescent cell antigen, which acts as a specific signal for cell disposal by initiating the binding of IgG autoantibody and subsequent removal by phagocytes (Kay *et al*., 1994).

Recently, deep sequencing unraveled that tRNA, rRNA, snoRNA, and YRNA can undergo processing into smaller RNA molecules (Rutjes *et al*., 1999; Rother & Meister, 2011; Sobala & Hutvagner, 2011). These new small RNAs are being noticed only now because most studies focused on sequencing reads derived from miRNAs. Accumulating evidence suggests that these RNA fragments are functional in physiological and pathological conditions (Rutjes *et al*., 1999; Haussecker *et al*., 2010; Anderson & Ivanov, 2014). In particular, tRNA halves have been described in the cytoplasm of stressed cultured cells where they have been reported to function in key biological processes, that is, translation and apoptosis (Ivanov *et al*., 2011; Anderson & Ivanov, 2014) and may be involved in human diseases including cancer, neurodegeneration, and infection [reviewed in Anderson & Ivanov (2014)]. More recently, we and others have reported that tRNA halves circulate in serum as abundantly as miRNAs (Meiri *et al*., 2010; Dhahbi *et al*., 2013b). The tRNA halves are mostly derived from the 5′ end of tRNAs and are not contained in exosomes or microvesicles, but circulate as particles of 100–300 kDa, indicating they may be actively secreted through an RNA‐binding protein‐dependent pathway. More interestingly, we have shown that the expression of tRNA halves is confined to the hematopoietic and lymphoid tissues in mice (Dhahbi *et al*., 2013b), which suggests that immune cells release tRNA halves into the circulation. As tRNA halves are expressed under physiological conditions at the whole organism level in immune tissues and concurrently circulate in serum, it is tempting to speculate that circulating tRNA halves mediate cell‐to‐cell communication mechanisms analogous to those involving the release, transport, and delivery of miRNAs from donor to recipient tissues. Unlike circulating miRNAs, which seem to be secreted by all types of peripheral tissues, circulating tRNA halves may function as systemic immune signaling molecules between various immune tissues, and/or from immune to nonimmune tissues. It remains to be demonstrated whether tRNA halves can exert immune‐related functions upon uptake by recipient cells in peripheral tissues.

We have previously found that mouse serum levels of specific subtypes of 5′ tRNA halves change markedly with age, and these changes can be attenuated by CR (Dhahbi *et al*., 2013b). Likewise, in this study, alterations in mouse circulating levels of 5′ tRNA halves derived from specific tRNA isoacceptors were associated with aging and dwarfism. Interestingly, the circulating levels of 5′ tRNA halves derived from tRNA‐His(GTG) were found to be increased with age here and in a previous study (Dhahbi *et al*., 2013b). The circulating levels of 5′ tRNA halves derived from tRNA‐Cys(GCA) and tRNA‐Lys (CTT) were also found to decrease with age in both studies. Notably, these age‐associated changes were mitigated by dwarfism as observed here and by CR as reported previously (Dhahbi *et al*., 2013b). Both, CR and dwarfism, have been shown to counter the molecular and biological markers of aging including alterations in gene expression study (Tsuchiya *et al*., 2004; Menon *et al*., 2014). The well‐characterized function of 5′ tRNA halves is the stress‐induced inhibition of translation initiation in cultured cells (Ivanov *et al*., 2011). It remains to be determined whether 5′ tRNA halves contribute to age‐associated dysfunctions and whether interventions such as dwarfism and CR exert their anti‐aging effects through regulation of cellular and extracellular levels of 5′ tRNA halves. However, our findings strongly suggest that the circulating levels of 5′ tRNA halves may have a role, whether as markers or effectors, in the manifestations of aging and in the anti‐aging effects of dwarfism and CR.

By implementing a statistical approach that models miRNA expression levels as a function of the genotype and the interaction between genotype and age, we are able to identify significant genotypic effects (i.e., differences in the expression levels of specific sncRNAs) that are age dependent. This approach, akin to adjusting clinical data for confounding variables, evaluates the effect of the interaction between genotype and age independently of individual effects of the genotype (the latter are not necessarily associated with aging directly but could occur due to the multiple endocrine abnormalities in the df/df model). In other words, we can detect the interaction effect once the independent effect of genotype by itself has been removed. This approach improves the odds of identifying genes that impact aging rather than some other process or characteristic (Swindell, 2007; Kent *et al*., 2012). There is a caveat in our experimental design, which due to the lack of a standardized scale of biological age equivalence between dwarf mice and normal mice considered animals of the same chronological age for each genotype. Normal and df/df mice of the same chronological age do not necessarily represent the same biological age (i.e., df/df mice may represent a younger biological age at an equivalent chronological age compared to normal mice; Flurkey *et al*., 2001). However, by focusing on GbA interaction effects, we inherently reduce the risk for biological misinterpretation.

In conclusion, we have shown for the first time that there is a signature of circulating miRNAs halves modulated by age in the long‐lived Ames dwarf mice. The miRNAs follow four different patterns of GbA interaction, which are mostly positive in df/df mice. Both, gene enrichment analysis of predicted targets and previous studies of target validation, support the involvement of these miRNAs in several biological processes and age‐related diseases. Our findings suggest that the extended lifespan in the df/df mice appear to be regulated, at least in part, by miRNAs that act through CR‐like and/or CR‐independent mechanisms. Futures studies will aim to identify the tissues releasing these GbA miRNAs into the circulation, as well as the target tissues and their specific functional roles in aging processes.

## Materials and methods

### Blood collection and serum preparation

Normal (N) and Ames dwarf (df/df) mice were bred and maintained under temperature‐ and light‐controlled conditions (22 ± 2 °C, 12‐h light/12‐h dark cycle). All mice used in the study were females. Serum was collected by cardiac puncture from 5 to 6 months old (young; 5 N and 5 df/df mice) and from 21 to 22 months old (old; 5 N and 5 df/df mice).

### RNA isolation and small RNA library construction

Isolation of total RNA, including small RNA, was performed with the miRNeasy kit (#217004; Qiagen, Hilden, Germany) according to the manufacturer's protocol with the following alterations: 1 mL of Qiazol reagent was mixed with 0.2 mL of serum, the entire aqueous phase was loaded onto a single column from the MinElute Cleanup Kit (#74204; Qiagen), and RNA was eluted in 20 μL of RNase‐free water. One‐fourth (5 μL) of the RNA isolated from each serum sample was used to construct sequencing libraries with the Illumina TruSeq Small RNA Sample Prep Kit (#RS‐200‐0012; Illumina, San Diego, CA, USA), following the manufacturer's protocol. Briefly, 3′ and 5′ adapters were sequentially ligated to small RNA molecules and the obtained ligation products were subjected to a reverse transcription reaction to create single stranded cDNA. To selectively enrich fragments with adapter molecules on both ends, the cDNA was amplified with 15 PCR cycles using a common primer and a primer containing an index tag to allow sample multiplexing. The amplified cDNA constructs were gel purified and validated by checking the size, purity, and concentration of the amplicons on the Agilent Bioanalyzer High Sensitivity DNA chip (#5067‐4626; Genomics Agilent, Santa Clara, CA, USA). The libraries were pooled in equimolar amounts and sequenced on an Illumina HiSeq 2000 instrument to generate 50‐base reads.

### Bioinformatics and statistics analyses of circulating small RNA sequencing reads

#### Alignment and annotation of sequencing reads

Sequencing reads were preprocessed with FASTX‐Toolkit (http://hannonlab.cshl.edu/) to trim the adaptor sequences and discard low‐quality reads. The filtered reads were mapped to the mouse genome (GRCm38/mm10) with Bowtie version 0.12.8 (Langmead *et al*., 2009) using the ‘end‐to‐end k difference (−v)’ alignment mode and allowing up to two mismatches. In addition, this mode of alignment was combined with options (−k 1 – best) that instructed Bowtie to report only the best alignment if more than one valid alignment exists. Annotation analysis of the mapped sequencing reads was performed with BEDTools (Quinlan & Hall, 2010) using noncoding RNAs (e.g., rRNA, snRNA, scRNA, and snoRNA) from Ensembl release 70 for Mus musculus (GRCm38/mm10 assembly), miRNAs from miRBase 20 (mirbase.org), and tRNAs from the Genomic tRNA Database (gtrnadb.ucsc.edu; Chan & Lowe, 2009).

#### Generation of the expression values of circulating tRNA‐derived small RNAs

The Bowtie alignment files obtained as described above were analyzed with BEDTools (Quinlan & Hall, 2010) to generate expression data by counting the reads that align to tRNA genes in the Genomic tRNA Database (Chan & Lowe, 2009).

#### Detection of miRNAs and generation of their expression values with miRDeep2

Sequencing reads were analyzed with miRDeep2 (Friedlander *et al*., 2012), which is probabilistic algorithm based on the miRNA biogenesis model and designed to detect miRNAs from deep sequencing reads. Briefly, miRDeep2 removes the 3′ adapter sequence and discards reads shorter than 18 nucleotides, before aligning them to the mouse mm10 genome. Only reads that map perfectly to the genome five or less times are used for miRNA detection. For the purpose of analyzing the sequenced miRNAs, the known miRNA input was from miRBase v.21 (Kozomara & Griffiths‐Jones, 2014), and Homo sapiens was designated as the related species. The miRDeep2 algorithm uses a miRNA biogenesis model to detect known miRNAs and discover novels miRNAs. It aligns reads to potential hairpin structures in a manner consistent with Dicer processing and assigns scores that measure the probability that hairpins are true miRNA precursors; it also estimates the expression levels of the identified miRNAs.

#### Differential expression analysis of the circulating small RNAs

Expression values of miRNAs and tRNA‐derived small RNAs obtained as described above were analyzed with the Bioconductor package edgeR (Robinson *et al*., 2010) to detect statistically significant changes in the levels of circulating small RNAs between the various experimental groups. The algorithm of edgeR uses the negative binomial model to measure differential gene expression. Expression values were normalized for the libraries size with the trimmed mean of *M*‐values method followed by estimation of the datasets dispersion. First, the overall dispersion was calculated by the function estimateGLMCommonDisp() to estimate the overall level of biological variability and calculate the coefficient of biological variation (BCV). Then, the functions estimateGLMTrendedDisp() and estimateGLMTagwiseDisp() were used to estimate genewise dispersion allowing a possible trend with average count size as recommended for experiments with multiple factors. The function plotBCV() was used to generate a plot that displays the estimated dispersions (Fig. S1). A generalized linear model (GLM) representing the study design [two age groups (Young and Old) with two genotypes (Normal and Dwarf)] was fitted to the data to create a GLM ‘fit’. A data frame ‘targets’ describing the treatment conditions was created. ‘Young’ was set as a reference for the age factor, while ‘Normal’ was set as a reference for the genotype factor. The design matrix was setup with model.matrix(~Genotype + Genotype:Age, data = targets). This design allows to determine the effects of age in the normal genotype using glmLRT(fit,coef = 3) and in the dwarf genotype with glmLRT(fit,coef = 4). Coefficients 3 and 4 represent the contrasts ‘GenotypeNormal:AgeOld’ and ‘GenotypeDwarf:AgeOld’, respectively. The GbA interaction was determined by glmLRT(fit,contrast = c(0,0,−1,1)). The interaction represents the difference of changes between dwarf and normal genotypes with respect to age. *P*‐values were adjusted for multiple testing using the Benjamini and Hochberg method to control the FDR.

### Tissue‐specific miRNA enrichment analysis

To determine whether some specific tissues could have contributed miRNAs to the circulation, we conducted enrichment analysis of tissue‐specific miRNA signatures in our list of GbA miRNAs. MicroRNA sets known to be enriched in mouse tissues (i.e., heart and brain) and described in Guo *et al*. (2014) were used. Significance (*P*‐value) of the match between tissue‐specific miRNA signatures and the circulating GbA miRNAs was empirically estimated by performing one million simulations in the r environment.

### Prediction of miRNA target genes and their functional analysis

The databases miRBase (Kozomara & Griffiths‐Jones, 2014) and TargetScan (Lewis *et al*., 2005) from the R packages microRNA (http://www.bioconductor.org/packages/release/bioc/html/microRNA.html) and targetscan.Mm.eg.db (http://www.bioconductor.org/packages/release/data/annotation/html/targetscan.Mm.eg.db.html) were used to predict the genes that are potentially targeted by the differentially abundant miRNAs. Final lists of putative targets are generated by the intersection of the targets predicted from TargetScan and miRBase. The predicted genes were analyzed with the Functional Annotation Chart tool from DAVID (da Huang *et al*., 2009) to determine which KEGG pathways and GO biological processes are specifically enriched in the gene lists. This tool measures the similarities among KEGG pathways and GO terms based on the extent of the associated genes and calculates an enrichment *P*‐value using Fisher's exact test. The enriched GO terms were further filtered to keep only GO biological processes with Benjamini multiple correction test value < 0.05. The filtered GO biological processes terms were processed with REViGO to remove redundant categories (Supek *et al*., 2011).

### Overrepresentation analysis of miRNA‐targeting events on predicted targets and miRNA–mRNA interaction network construction

We refer to this type of analysis as miRNA overtargeting analysis. Statistical hypergeometric tests adjusted for multiple comparison using the Benjamini–Hochberg correction were implemented in the r environment for comparison of observed proportions of GbA miRNA interaction events on each predicted target mRNA vs. expected proportions in the miRNA–mRNA interaction universe. This universe was build using the Conserved site context+ scores file downloaded from TargetScan Mouse (Release 6.2) at http://www.targetscan.org/cgi-bin/targetscan/data_download.cgi?db=mmu_61. For each predicted target mRNA, the number of interacting GbA miRNAs was calculated and the proportion to the total number of GbA miRNAs compared to the proportion of targeting miRNAs in the interaction universe. This comparison is used to infer whether an mRNA is overtargeted by GbA miRNAs (i.e., the mRNA interacts with a significantly larger number of miRNAs than it is expected by chance). This could indicate redundant regulation by or potential combinatorial action of miRNAs. To additionally enrich the list of putative overtargeted genes for biological relevance, significantly overtargeted genes (*P* < 0.05, FDR < 0.10) were analyzed with the Functional Annotation Clustering tool from DAVID Bioinformatic Database. This tool ranks the biological significance of gene groups based on overall EASY scores of all enriched annotation terms (da Huang *et al*., 2009). The clustering function measures the co‐association among the enriched annotation terms to group the similar, redundant, and heterogeneous annotation contents from the same or different resources into annotation groups. After clustering, the tool reports the most enriched clusters at the level of stringency selected. We used the highest stringency setting to detect significantly enriched annotation clusters with *P* < 0.05 and FDR < 0.25. The subsets of overtargeted genes enriched in the select functional clusters were then used to filter the list of interactions between GbA miRNAs and overtargeted mRNAs. This double‐filtered subset of relevant interactions (accounting for miRNA overtargeting and functional annotation enrichment) was used to build a miRNA–mRNA interaction subnetworks using Cytoscape 3.0.2 software (Shannon *et al*., 2003). The implementation of multiple independent biologically relevant filtering steps in our functional discovery strategy adds confidence to the eventual interaction network build.

## Funding

This research study was supported by National Institutes of Health (NIH)/National Institute on Aging (NIA; R01AG032290).

## Conflict of interest

None declared.

## Supporting information


**Fig. S1** Plot displaying the dispersion estimates. The tagwise dispersions are plotted against log2‐CPM.
**Fig. S2** Visualization of significantly enriched Gene Ontology (GO) Biological Processes associated with transcripts putatively targeted by the miRNAs whose serum abundance increases with age in normal mice but remains unchanged in df/df mice (pattern A).
**Fig. S3** Visualization of significantly enriched Gene Ontology (GO) Biological Processes associated with transcripts putatively targeted by the miRNAs whose serum abundance decreases with age in normal mice but remains unchanged in df/df mice (pattern B).
**Fig. S4** Visualization of significantly enriched Gene Ontology (GO) Biological Processes associated with transcripts putatively targeted by the miRNAs whose serum abundance decreases with age in normal mice but increases in df/df mice (pattern C).
**Fig. S5** Visualization of significantly enriched Gene Ontology (GO) Biological Processes associated with transcripts putatively targeted by the miRNAs whose serum abundance does not change with age in normal mice but increases in df/df mice (pattern D).

**Table S1** Circulating miRNAs exhibiting significant GbA in df/df and N mice, 0.10<FDR<0.38.

**Table S2** Gene Ontology enrichment analysis of transcripts putatively targeted by the miRNAs whose serum abundance increases with age in normal mice but remains unchanged in df/df mice (pattern A). (a) KEGG pathways enrichment analysis of transcripts putative targeted by the miRNAs whose serum abundance increases with age in normal mice but remains unchanged in df/df mice (pattern A).

**Table S3** Gene Ontology enrichment analysis of transcripts putatively targeted by the miRNAs whose serum abundance decreases with age in normal mice but remains unchanged in df/df mice (pattern B). (a) KEGG pathways enrichment analysis of transcripts putative targeted by the miRNAs whose serum abundance decreases with age in normal mice but remains unchanged in df/df mice (pattern B).

**Table S4** Gene Ontology enrichment analysis of transcripts putatively targeted by the miRNAs whose serum abundance decreases with age in normal mice but increases in df/df mice (pattern C). (a) KEGG pathways enrichment analysis of transcripts putative targeted by the miRNAs whose serum abundance decreases with age in normal mice but increases in df/df mice (pattern C).

**Table S5** Gene Ontology enrichment analysis of transcripts putatively targeted by the miRNAs whose serum abundance do not change with age in normal mice but increases in df/df mice (pattern D). (a) KEGG pathways enrichment analysis of transcripts putatively targeted by the miRNAs whose serum abundance do not change with age in normal mice but increases in df/df mice (pattern D).

**Table S6** Overtargeting analysis including 729 overtargeted genes.

**Table S7** Functional annotation analysis of overtargeted genes.

**Table S8** Circulating 5′ tRNA halves exhibiting significant genotype‐by‐age interaction.
