# Supplementary material for: Circulating microRNA signature of genotype‐by‐age interactions in the long‐lived Ames dwarf mouse
Source: Aging Cell. 2015 Jul 14;14(6):1055–66. doi: 10.1111/acel.12373 (PMC4693471; doi:10.1111/acel.12373)

**Figure S1**. Plot displaying the dispersion estimates. The tagwise dispersions are plotted against log2-CPM.

**Figure S2.** Visualization of significantly enriched Gene Ontology (GO) Biological Processes associated with transcripts putatively targeted by the miRNAs whose serum abundance increases with age in normal mice but remains unchanged in df/df mice (pattern A). DAVID was initially used to carry out the enrichment analysis of the gene lists, and subsequently REVIGO was used to remove redundancy and plot non-redundant GO terms as two-dimensional scatter plot. Similar GO terms (depicted by circles) cluster together closer than unrelated ones. Circle size (log size; legend in upper right-hand corner) is proportional to the frequency of a respective GO term in the Uniprot database used as background; circles of more general terms are represented by larger size. Circle color reflects the significance of GO term enrichment; it indicates the log10 of the p-value derived from DAVID analysis. Ends of the red and blue colors in the upper right-hand corner depict lower and higher log10 p-values respectively. GO terms representative of clusters are labeled; the complete list of the non-redundant GO Biological Process generated by the successive DAVID and REVIGO analyses are listed in Tables S1-S4.

**Figure S3.** Visualization of significantly enriched Gene Ontology (GO) Biological Processes associated with transcripts putatively targeted by the miRNAs whose serum abundance decreases with age in normal mice but remains unchanged in df/df mice (pattern B). See description in Figure. S1.

**Figure S4.** Visualization of significantly enriched Gene Ontology (GO) Biological Processes associated with transcripts putatively targeted by the miRNAs whose serum abundance decreases with age in normal mice but increases in df/df mice (pattern C). See description in Figure. S1.

**Figure S5.** Visualization of significantly enriched Gene Ontology (GO) Biological Processes associated with transcripts putatively targeted by the miRNAs whose serum abundance does not change with age in normal mice but increases in df/df mice (pattern D). See description in Figure. S1.


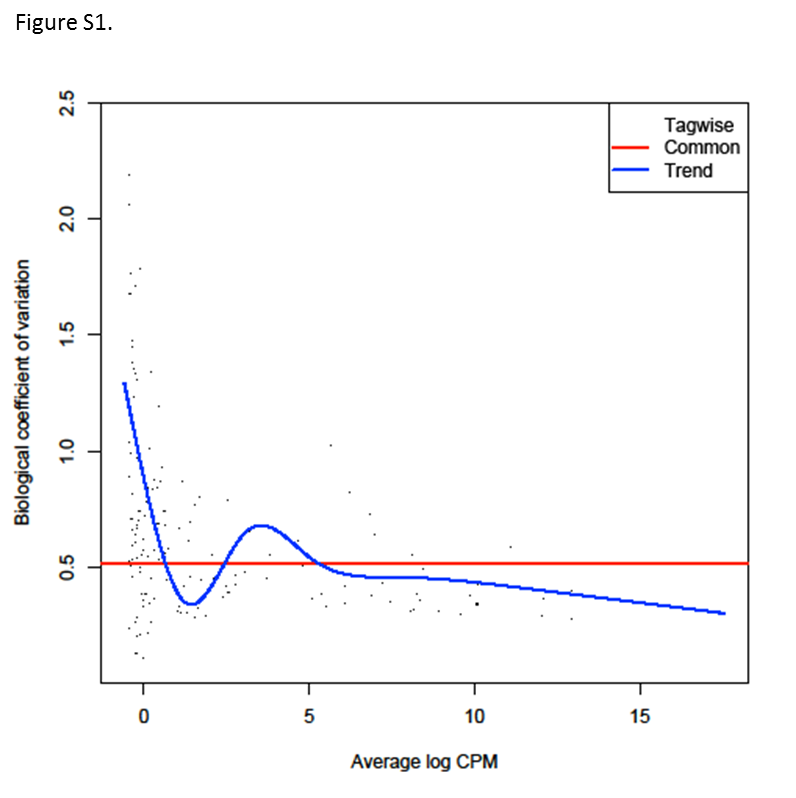


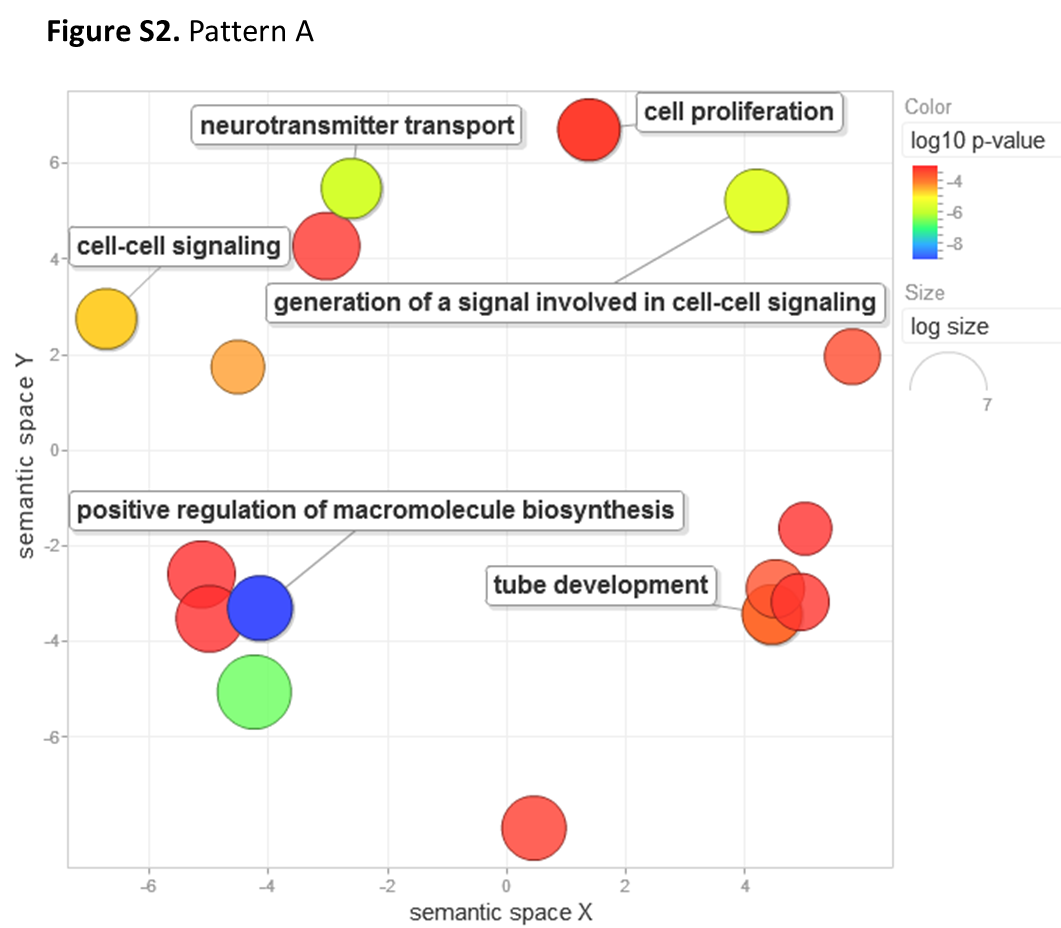


**
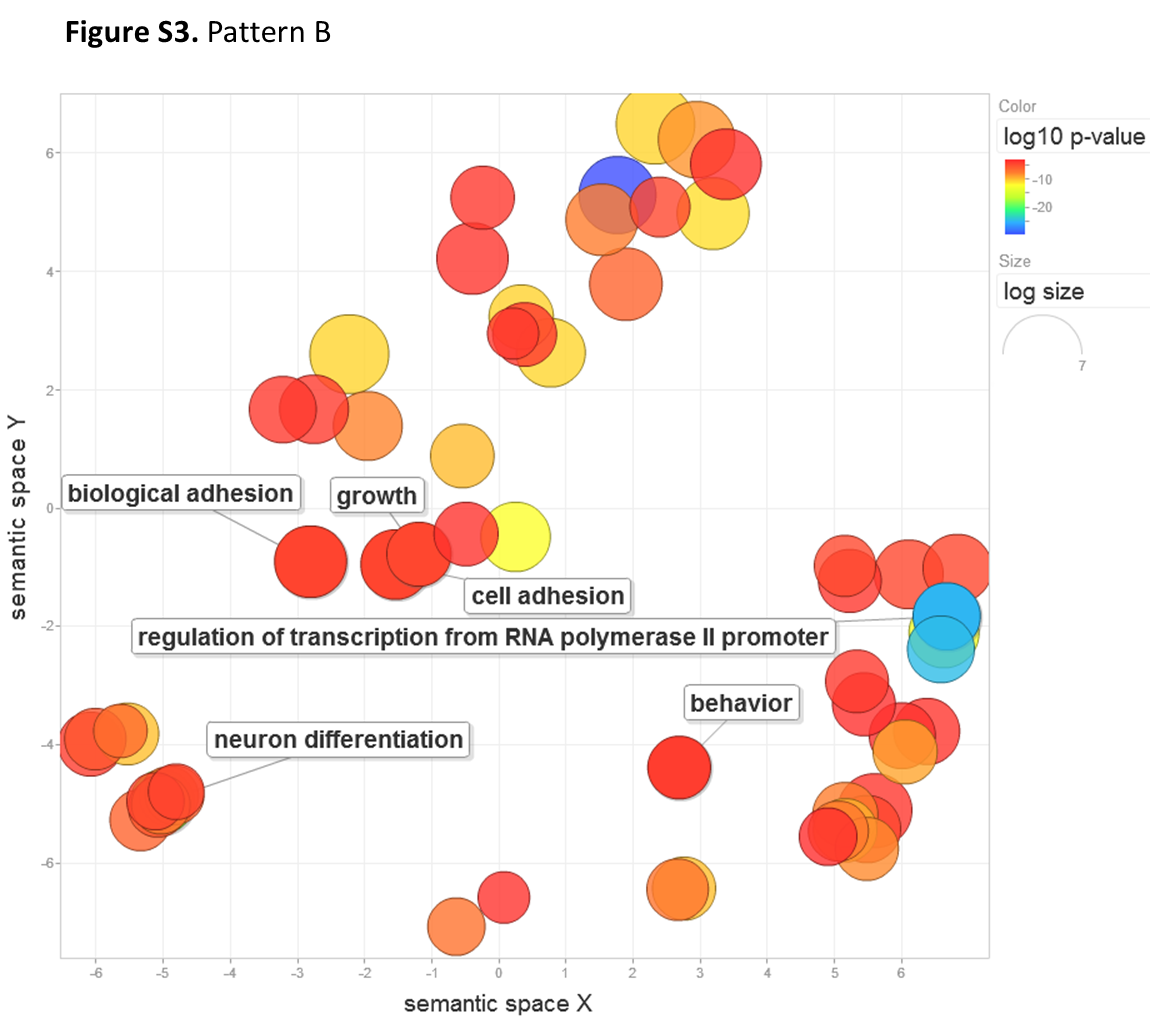
**


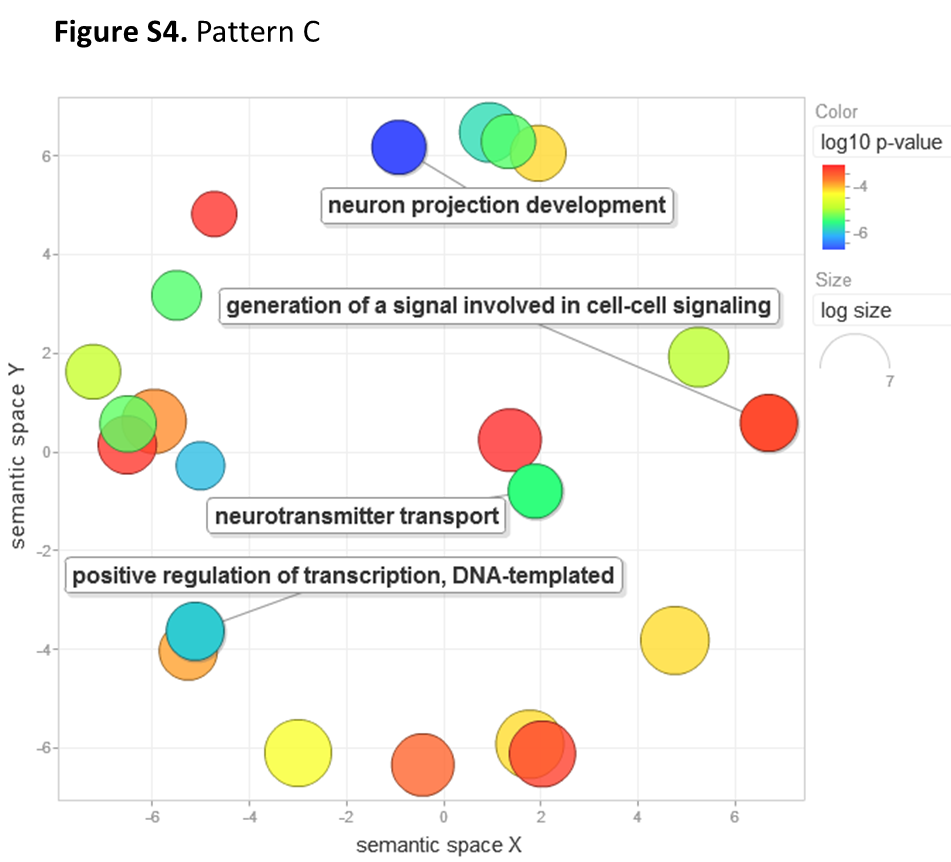


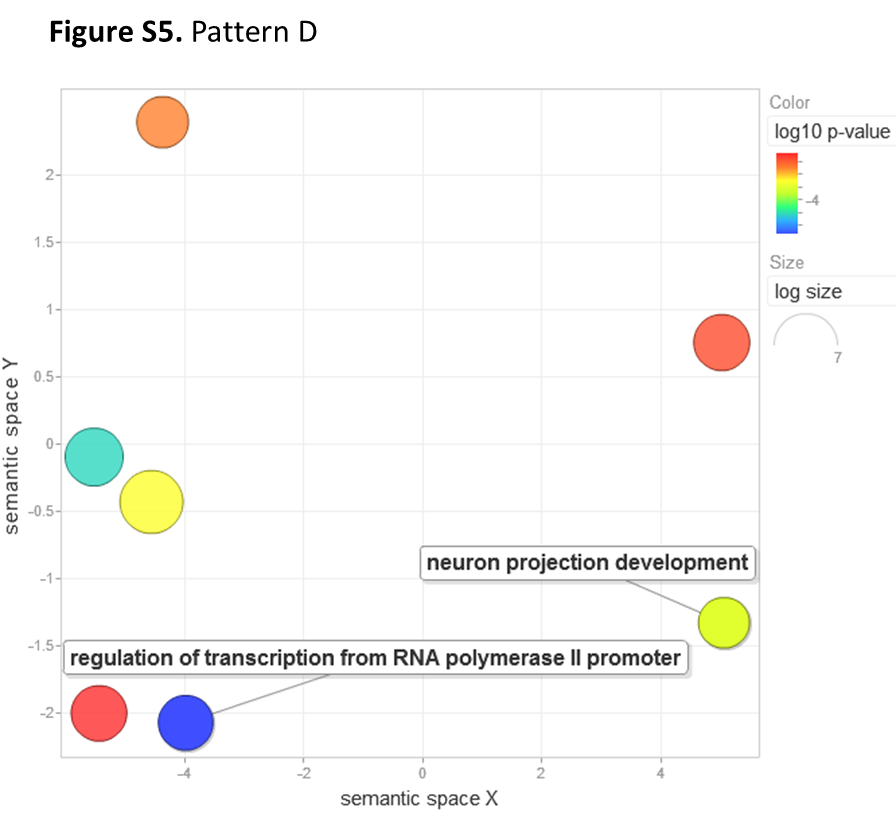

Supplement: Supplementary file 1 — Fig. S1 Plot displaying the dispersion estimates. The tagwise dispersions are plotted against log2‐CPM. Fig. S2 Visualization of significantly enriched Gene Ontology (GO) Biological Processes associated with transcripts putatively targeted by the miRNAs whose serum abundance increases with age in normal mice but remains unchanged in df/df mice (pattern A). Fig. S3 Visualization of significantly enriched Gene Ontology (GO) Biological Processes associated with transcripts putatively targeted by the miRNAs whose serum abundance decreases with age in normal mice but remains unchanged in df/df mice (pattern B). Fig. S4 Visualization of significantly enriched Gene Ontology (GO) Biological Processes associated with transcripts putatively targeted by the miRNAs whose serum abundance decreases with age in normal mice but increases in df/df mice (pattern C). Fig. S5 Visualization of significantly enriched Gene Ontology (GO) Biological Processes associated with transcripts putatively targeted by the miRNAs whose serum abundance does not change with age in normal mice but increases in df/df mice (pattern D). [file ACEL-14-1055-s001.docx]
